# Informed Consent in Asymmetrical Relationships: an Investigation into Relational Factors that Influence Room for Reflection

**DOI:** 10.1007/s11569-016-0262-5

**Published:** 2016-05-24

**Authors:** Shannon Lydia Spruit, Ibo van de Poel, Neelke Doorn

**Affiliations:** Department of Values, Technology and Innovation, Delft University of Technology, Delft, The Netherlands

**Keywords:** Informed consent, Nanomaterial risks, Relational autonomy, Room for reflection, Interpersonal relationships

## Abstract

In recent years, informed consent has been suggested as a way to deal with risks posed by engineered nanomaterials. We argue that while we can learn from experiences with informed consent in treatment and research contexts, we should be aware that informed consent traditionally pertains to certain features of the relationships between doctors and patients and researchers and research participants, rather than those between producers and consumers and employers and employees, which are more prominent in the case of engineered nanomaterials. To better understand these differences, we identify three major relational factors that influence whether valid informed consent is obtainable, namely dependency, personal proximity, and existence of shared interests. We show that each type of relationship offers different opportunities for reflection and therefore poses distinct challenges for obtaining valid informed consent. Our analysis offers a systematic understanding of the possibilities for attaining informed consent in the context of nanomaterial risks and makes clear that measures or regulations to improve the obtainment of informed consent should be attuned to the specific interpersonal relations to which it is supposed to apply.

## Introduction

The notion of informed consent has major significance when dealing with the risks associated with medical treatment and experimentation. The main idea behind informed consent is that individuals should be able to make their own knowledgeable and voluntary decisions concerning their exposure to potential dangers, thereby emphasizing the importance of individual autonomy and responsibility for balancing risks and benefits. In this paper, we discuss the application of informed consent to engineered nanomaterial risks. To do justice to the different social context in which nanomaterial risks emerge, we explore how social relations influence the validity of informed consent.

Toxicologists, risk assessors, and environmental scientists have not yet reached consensus on the alleged hazardous effects of newly engineered nanoparticles and nanostructured materials (from now on “nanomaterials”^1^). Innovation in fields such as material sciences, chemistry, and physics has led to the possibility to create and manipulate matter on the nanoscale. This has led to the production of nanomaterials with economically promising new traits such as a higher reactivity, different polarity, and increased mobility. However, the identification and evaluation of these materials is problematic due to a general lack of knowledge about them and of how they interact with the environment. Furthermore, limitations in measurement techniques have made formulating occupational exposure limits difficult [1]. Nevertheless, caution is advised, especially in the occupational context, due to the potential risk to human health and safety [2–4].

In response to this uncertainty about hazards, several authors have suggested that informed consent may be applied to decisions on the desirability of new nanomaterials. It has been argued that nanomaterials are experimental in the sense that the impact of these risks may become fully clear only after these new materials have been introduced into society [5, 6]. Although there is much uncertainty about the risks posed by nanomaterials, they are now regularly introduced into the environment and society [7, 8]. In that respect, informed consent may be an interesting model to judge the acceptability of such social experiments with nanomaterials. And indeed, proponents of informed consent in the context of nanomaterials argue that it would be better to obtain some form of consent from citizens, allowing them to decide whether they are willing to be exposed to the risks posed by these technological products. Shrader-Frechette considered this one of the primary duties of government in regulating nanomaterials, arguing that they must “… help citizens attain their rights to free informed consent to nano-related risks [9, pp 49].” Similar suggestions were made by Jacobs and colleagues [6], who considered the absence of genuine consent in relation to the risks posed by nano titanium dioxide to be a topic of ethical concern.

Informed consent is an established principle to deal with risks in the field of medicine [10–12], but its translation to the use of nanomaterials is not straightforward. An objection to the use of informed consent for nanomaterials may be the uncertainty that accompanies these materials. Consent cannot be informed if it has no solid knowledge base, something that seems to be exactly missing in the case of nanomaterials. Without sufficient knowledge about what the risks of nanomaterials are, it may be hard to balance them against their alleged benefits and come to valid informed consent. Each medical product will have gone through extensive testing before it enters the market, whereas risk assessment of nanomaterials is hardly sufficient to cover all products developed in this emerging technological field [13]. However, at closer examination, the differences are less clear. After introduction to the market, many drugs present unexpected side effects and unanticipated interactions [14], making informed consent in a treatment context sensitive to uncertainty as well. Participants in clinical trials are confronted with even more uncertainty when they decide to be subjected to experimental drugs. Therefore, uncertainty accompanying nanomaterials is in our view not a principled reason to refrain from the implementation of informed consent in the governance of nanomaterials. Rather, dealing with uncertainty requires an open dialogue about potential side effects and the limitations of knowledge thereof that is central in informed consent procedures.

Nevertheless, the context in which informed consent is traditionally used and developed—namely the medical field—differs from the context in which most nanomaterials are used [15]. Drug use and medical research are more highly regulated than the production and application of nanomaterials that are characterized by regulatory gaps [16–18]. Additionally, while the need for medical treatment and the development of new drugs is often taken for granted, the benefit of nano-enabled products is less evident [19]. Furthermore, in medical treatment and research, the application of potentially hazardous materials is relatively contained; medicines are administered to individual humans, whereas nanomaterials may be used in a variety of consumer products and settings ranging from sports equipment, building materials, to shoe polish [8, 20]. Unlike individual risk-taking in medical contexts, exposure to nanomaterials is more collective in nature which renders informed consent unsuitable [21]. However, this is not the case for all exposure to risky nanomaterials. In this paper, we look at individual market transactions and workplace exposure to risky nanomaterials both of which are situations that Hansson identifies as those in which informed consent could potentially be obtained [21].

Our focus is on one specific difference between the medical context of treatment and research and the wider context in which nanomaterials are applied: the relationships between those who produce risks and those who are exposed to them. We assess how differences in relationships may affect the obtainment of informed consent. We look at the risks posed by nanomaterials and the main challenges for achieving informed consent in two settings in which nanomaterials are handled. Workers are among the first people to be exposed to new nanomaterials, and their relationship with their employer influences the voluntariness of that exposure. Although a consumer’s decision to buy a product containing nanomaterials may be considered a form of consenting to nanomaterial risk, the quality of the relationship with the producer—for example, whether the relationship is transparent or more distant—influences the transfer of information about risks. By exploring the characteristics of the producer–consumer and the employer–employee relationship, and the opportunities these relationships provide to obtain informed consent as well as the constraints they place on doing so, we are able to say more about how informed consent may, or may not, be obtained for the risks posed by new technologies.

We build on the idea that, in practice, the qualities of interpersonal and social relationships^2^ influence the obtainment of valid informed consent. Differences in power, knowledge, and influence may require a different interpretation or operationalization of informed consent in non-traditional contexts. Several authors acknowledge the importance of the relational setting for informed consent [22–26]. A detailed analysis of exactly which characteristics of relationships influence the obtainment of valid informed consent outside of treatment or research relationships is, however, lacking. Recognizing the relational influences on informed consent may assist us either to conclude that informed consent is not feasible in the context of technological risks or to adapt the notion of informed consent to certain contextual constraints while maintaining its normative core.

In this paper, we first discuss informed consent in terms of its various components: competence and voluntariness, the transfer and understanding of information, and the ability to make an individual choice. We then expand this view by presenting informed consent as functioning in and being the product of a particular relationship. We draw from academic literature to identify which aspects of relationships (mainly in medical or research settings) are known to affect informed consent. Based on these findings, we present a more detailed characterization of the relationships between producer and consumer and between employer and employee. We conclude with a discussion about how best to take such relational aspects into account when conceiving of informed consent in the context of the risks posed by nanomaterials.

## Informed Consent in Context

The practice of informed consent has its roots in the medical context. Informed consent refers to the process of getting permission before a healthcare intervention can be conducted on a person. It is one of the fundamental ethical principles in both clinical treatment (medical ethics) and clinical research (research ethics). Informed consent functions within a broader regulatory framework as a legal safeguard to protect the patient’s individual autonomy [27]. However, this paper focuses on informed consent as an answer to the moral intuition that many people share, namely that people have the right to know what risks they are being subject to and the right to freely choose which risks they are willing to be subject to and which ones they do not wish to be subject to.

In medical and research ethics, attempts have been made to define the elements that constitute informed consent. Although there are different classifications, the following elements are generally considered to capture the basic notion of informed consent: a “threshold component,” an information component, and a consent component [28–30]. The threshold component indicates the preconditions for informed consent, including the competence of patients and research participants for independent decision-making and the voluntariness (i.e., the absence of coercion) of their decisions. The information component comprises standards for disclosure of information by doctors, as well as patients’ understanding of that information. Discussions concerning the consent component include the conditions under which a decision can be considered valid. For example, is a procedurally correct decision sufficient or does it refer to “an individual’s *autonomous authorization* of a medical intervention or of participation in research?” [italics in original; 31, pp 78].

These components of informed consent set conditions for reasoning capacities, information transfer, and control thereby ensuring individual capacity to autonomously choose medical procedures or research participation. However, the literature on the various components of informed consent reveals that the ideal case of a “purely autonomous individual”—one who independently informs herself and makes an independent decision based on her own assessment of the desirability of her exposure to risks—often does not exist in practice. For Buehler [32], this is a case of “wishful thinking,” since patients primarily make medical decisions in close consultation with their doctors. The professional is also strongly involved in the information component as she has to ensure that the patient has received sufficient information and reached a full understanding to make an autonomous decision [31]. But also framing effects or informational manipulation may lead to patients being influenced by doctors when information is presented or framed in such a way that the patient is directed toward a particular choice thereby threatening the possibility of voluntariness [33, 34]. Such influence is not, of course, only internal to the doctor–patient relationship. Third parties, social expectations, and pressure may also pose a threat to the validity of informed consent [35]. For example, expectations about gender roles have been shown to influence women who consented to gynecological interventions even though they did not really agree with the medical procedures [36].

Most, if not all, decisions are not the mere result of individual reasoning based on individual capacity but are shaped by a social context consisting of personal, family, and professional relationships. This observation has led people to argue that we should conceive of autonomy not as an individual capacity but as a relational notion [37], shifting attention to the interpersonal dynamics that underlie individual decision-making.

Certain kinds of relationships may impede with individuals’ ability to make independent decisions, whereas others facilitate and foster voluntary and informed decision-making. This goes beyond direct interference. Proponents of this notion of relational autonomy have argued that even our conception of autonomous choice is itself shaped by, and is the product of, a specific social context in which we live. Relationships with parents, teachers, friends, etc. shape our identity, our capacities, and our understanding of ourselves as autonomous individuals [38]. If socialization is oppressive, this may be detrimental to the ability of people to reflect on their options. McLeod and Sherwin for example argue that being members of oppressed groups, such as immigrant communities, in the long run leads to reduced self-trust and can undermine group members’ capacities for autonomous choice in medical settings [39].

If we take seriously the claim that social relationships shape autonomy to a great extent, then we need a conception of informed consent that is sensitive to the relational context in which it functions. Therefore, the remainder of this paper will be a first step in developing a relational approach to informed consent for nanomaterial risks. We maintain that the quality of informed consent can be assessed not only in terms of individual capacity but also as the product of a specific relationship and the characteristics of the relationship in which it functions.

A key notion here is reflection: the validity of informed consent depends on the room for individuals to reflect on the influence of their surroundings and the quality of that reflection. Christman argues that as long as a person “… maintains the ability to adequately reflect on … [her] conditions and embrace them … we should continue to label her autonomous” [40, pp. 155]. This means that compliance with a social setting, with its norms and values, or even obedience to others, can be considered autonomous as long as an individual makes this choice after having reflected on it. This, of course, presupposes that an individual has an actual choice and the liberty to make that choice [41]. Reflection cannot compensate for an absence of alternatives. However, we know that reflection itself is susceptible to socialization. The focus of our discussion here will not be on what makes up qualitatively good reflection and what influence socialization should or should not have; this goes beyond the scope of this paper. Nonetheless, our discussion will show that several characteristics of relationships can contribute positively to empowering individuals that may have been silenced or rendered powerless.

Indeed, we will develop a more procedural approach with a focus on the context of decision-making rather than a substantive approach that would define what autonomy actually entails and what type of behavior or thoughts are necessary for autonomous choice. Several authors have argued for something similar [25, 37] but not in the context of nanomaterial usage. In the following, we expand this procedural relational approach to informed consent with a view to informing a debate about the desirability of informed consent for dealing with nanomaterial risks. The next section discusses a characterization of relationships in order to examine in more detail the ways in which relationships affect room for reflection and thereby the validity of informed consent in consenting to risks.

## Informed Consent Decisions in Different Types of Relationships

A range of relationships may be at play in informed consent decisions. Here, we model these relationships as an example of a dyadic relationship between a risk-imposer and a risk-bearer. The basic idea is that one party (the risk-imposer) introduces a risk that is borne by another party (the risk-bearer). At present, informed consent means that the risk-imposer is allowed to introduce a risk if, and only if, the risk-bearer has given her informed consent to the introduction of the risk. This is obviously a simplification of the various relationships that are at play in informed consent. However, this approach is helpful in establishing some of the structural differences between relationships in which informed consent has already been studied extensively—that is, those between doctor and patient and between researcher and research participant—and relationships in which informed consent is less studied, namely those between producers and consumers and between employers and employees and that are relevant to the case of exposure to nanomaterial risks.

This section proposes a framework for characterizing the relationship between risk-imposer and risk-bearer that is based on the literature on doctor–patient and researcher–research participant relationships. We also discuss how structural differences between relationships allow for more or less room for reflection and thereby influence the validity of informed consent. In the “Discussion” section, we use this framework to characterize the relationships between producers and consumers and between employers and employees.

### Ideal-Typical Relationships in Medicine and Research Settings

Although many authors emphasize the importance of relationships in arriving at autonomous decisions, they have largely left the nature of these relationships undiscussed. Nevertheless, some typologies have been proposed to capture the structural differences between the relationships in which informed consent decisions are made. The ideal types discussed in this section refer to various forms and dimensions of control between patients and doctors and investigators and subjects. In these models, actors exert influence at different levels and in different ways in relation to different expressions of autonomy.

Ezekiel and Linda Emanuel offer a well-known typology that distinguishes four models of the doctor–patient relationship [42]. The first, the “paternalistic” model, describes a doctor who is considered the patient’s guardian, treating the patient in a way that is deemed medically correct, with the patient simply assenting to the doctor’s decision. In the “informative” or “consumer” model, the doctor provides the patient with all the relevant information and leaves the decision-making to the patient. This model offers more autonomy than the paternalistic model as patients are seen as actively making decisions rather than simply following the doctor’s advice. There have been objections to this model, however, because it makes the role of the doctor too technical and lacks a “caring approach” [43]. This objection is overcome in the “interpretive” model, where the doctor helps the patient to reflect on her own values and decide what she wants. In this case, the doctor acts more as a consultant or therapist. Emanuel and Emanuel’s fourth model, the “deliberative” model, is more symmetrical: the doctor actively discusses treatment with the patient, who uses the doctor’s expert knowledge but also actively engages in her own treatment process.

The main distinguishing factor between the ideal types described by Emanuel and Emanuel seems to be the level of control over medical decisions (see Table 1). More paternalistic models leave little room for patient agency, whereas the interpretive and informative models emphasize patients’ individual freedom of choice. Even though some models, such as the informative model, ascribe a smaller role to doctors in the actual making of a decision, they may still be highly influential through the way they present information. These “framing effects” can be very powerful sometimes even creating anxiety and harming patients [44].

**Table 1:** The table summarizes the models of medical treatment relationships, based on the work of Emanuel and Emanuel [42]. The columns represent different ways of dividing responsibilities for medical decision-making. Even though some models, such as the informative model, ascribe a smaller role to doctors in making the decision, they may still highly influence it by presenting information in a particular way

	Paternalism	Informative/consumer	Interpretive	Deliberative
Who has control over the information?	Doctor	Doctor actively shares all information with patient	Doctor about medical information, patient about own values	Both share and exchange information, although doctor has medical expertise
Who makes the decision?	Doctor	Patient chooses; doctor has to accept patient’s preferences	Doctor helps patient as consultant to discover own preferences and values	Patient and doctor deliberate together, although the patient makes the final decision

Other ideal types that have been developed in research environments show that differences in the relationship between researcher and research participant are multidimensional and potentially asymmetrical in various ways. Philosopher Joan Cassel [45] developed a typology of research relationships to assess the appropriateness of ethical principles in those relationships (Table 2). She distinguished between research relationships in different fields showing how they vary in terms of the researcher’s power and control over the research setting and context as well as the direction of the research interaction. Experimental biomedical researchers have a lot more power and control over their research participants compared to anthropological field workers who use such methods as participant observation. In biomedical experimentation, researchers define the experimental setting and determine what the participant should do. This is much less the case in social sciences research in which the research participants have much more agency because the research takes place in their own social settings, and methods of participant observation require minimal interference by the researcher (for example [46]. Finally, the direction of interaction in research is a further important distinguishing factor. Participants in biomedical, psychological, and survey research have very limited interaction with the researcher (they basically provide answers and/or information), while in anthropological fieldwork, the participants often actively steer the research and have a much more equal role in communication. A limitation of this typology is that it does not specify what characteristics of relationships allow for more or less control or more or less power on both sides.

**Table 2:** An overview of influence and control in research relationships, adopted with minor adjustments from Cassel [45]

Dimension of control	Biomedical experimentation	Psychological experimentation	Survey research	Fieldwork (participant observation)
Investigators’ power as perceived by participants	High	High-medium	Medium-low	Equal
Investigators’ control over research setting	High	High-low	None	Negative control by participants
Investigators’ control over context of research	High	High	Medium-low	Equal
Direction of research interaction	One-way: investigator examines research participant	One-way: investigator examines research participant	Limited two-way: in the case of unstructured surveys (interviews), participant can interact more freely with investigator	Two-way: investigator and participant strongly influence each other

### Relational Factors that Influence Informed Consent

To clarify which characteristics of relationships allow the risk-bearer to be more autonomous, we need a more detailed conception of what constitutes these relationships. Therefore, this section presents empirical literature from bioethics, the social sciences, and law on the relational characteristics that influence informed consent practices or similar kinds of decisions. We look at three types of relational factors: (1) dependency, (2) personal proximity, and (3) shared interests. This information can provide us with insight into the relationship between informed consent and the social context of its application, in general, as well as deepen our understanding of which aspects of relationships may be supportive of or disruptive to relational autonomy.

#### Dependency

Asymmetry in expertise and knowledge concerning the risks associated with products and treatments makes risk-bearers dependent on risk-imposers for disclosure and an understanding of the information. Risk-imposers and risk-bearers often do not have the same information about the risks of an intervention or drug or about exposure to dangerous substances. According to Faden and Beauchamp [10], the main obstacle to informed consent in the medical context is informational manipulation which occurs when information is presented or framed in such a way that the patient is directed toward a particular choice [10, 47, 48]. This can be done because there are often great inequalities in the information available to patients and doctors—this is known as “informational asymmetry” [22]—with patients often depending on doctors for access to, and the interpretation of, risk information. Aside from dependency in the provision of information, there may also be a difference in the capacity of risk-bearers to understand the information provided by doctors and researchers [49], for instance due to illness [50, 51] or simply the patients’ lack of expertise.

Dependencies—such as informational asymmetry and risk-bearers’ dependency in understanding risk information—may give rise to situations in which the obtainment of informed consent is strongly influenced by the power relation between risk-imposer and risk-bearer. The existence of power is almost by definition relational: Power exists only by virtue of there being somebody to influence or control with that power. In medical practice, doctors can exert considerable influence over medical decisions by, for example, imposing their values. Coercion is not at all uncommon in medical practice [see for instance, 48, 52–54]. Furthermore, doctors are important gatekeepers for medical treatment and medical resources.

#### Personal Proximity

The ties between the risk-imposer and the risk-bearer have become increasingly closer in research and medical practice. According to Miller and Boulton [55], research relationships in the social sciences have become much more personal and conversations have become more dialogical. Researchers sometimes develop what are known as “fake friendships” with participants, immersing themselves in the field through participant observation resulting in very open contact.

A similar phenomenon can be seen in the medical field, where doctors and patients need common ground to discuss the course of treatment. For example, shared values are said to be very important in meeting patients’ physical and psychological needs [56]. It may also be important for doctors to become familiar with their patients’ frames of reference and values to understand why their patients turn to alternative treatments that, medically speaking, may not be the best choice. Additionally, close bonds also seem to positively influence information transfer and genuine understanding as a personal and trusting relationship positively influences the understanding of medical information [57].

Furthermore, the continuity of therapeutic and research ties may interfere with the validity of consent. Doctor–patient interactions can be seen as long-term commitments rather than as one-off decisions to arrive at a form of consent [22]. These relationships are built over time: a doctor monitors a patient’s health and may change the treatment regime as required. Something similar is seen in sociological and anthropological research. For example, in participant observation, there is an ongoing interaction between the researcher and the participant [58]. On the one hand, building strong and long-term bonds in the field is often inherent in being a good social researcher. On the other hand, individuals may feel pressured to make choices that maintain their relationship with, for example, health professionals thus threatening the voluntariness of their consent [26].

Therefore, informed consent procedures must achieve a balance between closeness and distance. Informed consent is often presented as an alienating, formalized practice that can be very impersonal and highly bureaucratized. For instance, according to Clarke [59], many informed consent procedures have become formalized decisions that may leave little room for questions and dialogue. Miller and Boulton [55] also observed a strong standardization of ethical and informed consent procedures in the social sciences. However, this does not always need to be an issue. For instance, in social science research, there is often no clear moment of initiation or involvement in a study [60]. Especially when using participant observation, a research participant is unlikely to be directly asked for her informed consent [24]. There seems to be no clearly demarcated moment for doing so and, in this respect, increasing formalization may assist the actual obtainment of informed consent. This indicates that maintaining the right distance is key to making informed consent decisions.

#### Shared Interests

The relationships in which informed consent decisions are made differ in the degree to which there is some sort of shared interest between the risk-imposer and the risk-bearer. This shared interest is most obvious in the treatment relationship between doctor and patient: Here, the doctor has a fiduciary duty toward the patient which means having to act according to the interests of the patient [22, 61]. Thus, a doctor must not only inform patients about risks and allow them to make a decision but also tell them about alternative treatments. This is not to say that patients’ interests are always entirely clear: Research has shown that there is strong variation between patients’ preferences in medical decision-making [62]. Patients may also form and shape their preferences during the decision-making process in line with the interpretative model of Emanuel and Emanuel [42] in which doctors help patients to discover and order their values to come to medical decisions. However, patients or their guardians may misinterpret the intentions of doctors due to the social status of medical institutions. For example, in academic hospitals, doctors are often also researchers who have an interest in high rates of participation in trials [63]. This has consequences for the obtainment of informed consent: Parents may misinterpret activities that are experimental as being therapeutic and think that the doctor is only acting in their child’s best interest. Something similar can be seen in the social sciences, where researchers often engage with politically loaded or sensitive topics. In these settings, researchers may be mistakenly perceived as advocates or activists, whereas their primary loyalty is to the academic community in providing a theoretically sound, objective account—though various forms of engaged anthropology seem to merge these two roles [64].

### Room for Reflection

As argued in the “Informed consent decisions in different types of relationships” section, relationships influence the room for reflection that is crucial for obtaining valid informed consent. The previous section discussed the characteristics of relationships in which informed consent decisions are made. Depending on their characteristics, relationships may offer different opportunities for reflection. For instance, in a relationship in which there is strong dependency because of informational asymmetry, risk-bearers have less capacity to genuinely reflect on their options. Strong personal bonds, on the other hand, may enhance reflection as is revealed in the deliberative model of the doctor–patient relationship. The existence of conflicting interests may prompt reflection as conflict enables us to see differences in the values that guide risk decisions.

The relational factors identified in the previous section enable us to evaluate the validity of informed consent from a relational perspective (see Table 3 for an overview) not only because these individual factors themselves influence the reflection of risk-bearers on the information and decisions with which they are confronted but also because these factors help the risk-bearer to reflect on her relationship with the risk-imposer: One can only be really autonomous by reflecting on the characteristics of the relationship one is in, and how those characteristics may influence the decision one makes.

**Table 3:** Relational factors that may influence the autonomy of informed consent or similar decisions

Factor	Evaluated in terms of:
Dependency	• Dependency on risk-imposer for information • Understanding dependency due to differences in level of education, knowledge, and cognitive abilities between risk-bearer and risk-imposer • Dominance of one actor’s view in decision • Existence of visible and invisible coercion
Personal proximity	• Level of anonymity, intensity if contact, and sharing of personal information • Relational continuity, duration of the contact (multiple meetings) • A balance in formalization/informalization of contact (e.g., as a result of standardization and bureaucratization)
Shared interests	• Similarity between the interests of the risk-bearer and those of the risk-imposer, as well as the extent to which one can rely on another to act in one’s interests • Existence of legal and moral duties of one party to protect the other party’s interest • Presence of commercial or other conflicting interests

## Characterizing Employer–Employee and Producer–Consumer Relationships

Relationships need to meet certain levels of independence, shared interests, and proximity to allow for reflection and the obtainment of valid informed consent. This section explores two relationships that are prominent in decisions concerning nanomaterial risk: the relationship between employer and employee and that between producer and consumer. We characterize these relationships in terms of the characteristics introduced in the previous section, that is, in terms of dependency, personal proximity, and the existence of shared interests. Next, we discuss what room for reflection these relationships offer.

### Relational Factors

The characterization we present describes the employer–employee and producer–consumer relationships, in general, in ideal-typical terms.

#### Dependency

The relationship between producers and consumers is often considered to be one in which the consumer is fairly independent. In an ideal market, market transactions are, by definition, similar to informed consent decisions: For a market transaction to be considered genuine, all decisions have to be made knowingly and willingly. Producers and consumers are fairly autonomous in their decision to partake in a specific market transaction: they can buy from or sell to whomever they choose. Nevertheless, in many cases, there may also be some form of dependency among consumers because, for example, there are not enough alternatives to make a free choice, or a producer has a monopoly on certain products (such as the near monopoly of Microsoft in the 1990s).

In ideal market transactions, both the producer and the consumer have complete information about the product and its possible hazards, which would suggest we can see it as a form of informed consent. In practice, of course, complete information is never available. In many jurisdictions, therefore, the producer has a legal duty to inform buyers about any known negative effects through labeling thus making the informational position of consumers somewhat similar to that of the patient in the doctor–patient relationship. One difference from the doctor–patient relationship, however, is that a producer is not expected to offer consumers information about alternatives that may better suit their needs—which would obviously be against the producer’s economic interests. Moreover, there is usually no personal contact between the risk-imposer (producer) and the risk-bearer (consumer), unlike in the doctor–patient relationship. This may make transferring knowledge of the risk more difficult, with the risk-imposer unable to verify whether the risk-bearer has really understood the risk. As a result, informational asymmetry threatens the risk-bearer’s opportunity to reflect on the desirability of being exposed to that risk. Also, consumers often have little or no say in design processes (despite all the participatory initiatives that have emerged in recent years), so in this respect, too, they very much depend on the efforts of producers to make less risky products.

The presence of free choice is much less obvious in working environments. The hierarchy in working environments means the employer is responsible for the working conditions of her employees: employees depend greatly on their employers to make the right decisions concerning exposure to risks, and employers are in many countries legally responsible for organizing a safe working environment. The individual choice of employees is limited, making this type of relationship rather asymmetrical in terms of power. It must be noted, though, that the academic environments in which nanomaterials are used and produced are typically characterized by more independence and self-determination in working practices.

#### Personal Proximity

The strength of ties in both employer–employee and producer–consumer relationships may vary considerably depending on the context. In some markets and for some products, there may be strong and enduring ties between producers and consumers; in other cases (e.g., consumer products that can be bought at a supermarket), the ties may be much looser: Consumers may be anonymous to producers and a personal bond may be absent. Employer–employee relationships are, in general, less anonymous as there is often interaction on a daily basis and these relationships usually last longer than producer–consumer relationships. Again, however, there may be quite some differences between employees who are hired on a temporary basis through an employment agency and those who spend their entire working lives with the same employer which leads to strong personal bonds between them.

When we focus on nanomaterials, the following ideal-typical picture arises. Companies that develop and use nanomaterials often employ skilled workers who, we assume, work there for at least a number of years making the ties between employer and employee stronger and distances (e.g., in hierarchy) easier to bridge. Although this may lead to shared interests (see also the following section), long-term bonds may also pose a risk and lead to self-sacrificing behavior by employees out of loyalty to an irresponsible employer or in order to hold on to a valuable job. Phenomena such as group think—a tendency to prefer harmony within the group over the right or best outcomes—suggest that stronger social ties may also diminish the space for reflection [65]. Similarly, we know that shared decision-making processes may lead to group polarization [66] where a groups comes to hold more extreme views than the individual members of the group held initially.

Market transactions are, in general, much more distant. As is the case in biomedical research settings, producers and consumers do not have contact on a regular basis, and any contact there tends to be relatively brief and instrumental. Furthermore, market transactions often go through sellers and re-sellers, leading to large distances between the risk-producer and the consumers who are exposed to the risks. This limits the opportunity for consumers to communicate with producers about the risks posed by new technological products with the contact rather unidirectional. This is especially the case for one-time buyers although some consumers are loyal to particular brands. The latter may be in a better position to make informed decisions because of this stronger connection.

#### Shared Interests

If we assume that producer–consumer relationships are guided by the market, then producer and consumer have a shared interest in the transactions they agree on, but apart from that, there need not be a shared interest. In comparison, employer–employee relationships seem to be characterized by stronger shared interests, as both have, at least in principle, a shared interest both in the performance of the company and in some minimally decent working conditions. Nevertheless, the history of labor movements suggests that these shared interests have not always been recognized and acted upon by the parties involved.

If we focus on nanomaterials, it is particularly interesting to look at the extent to which there is a shared interest in safety and protection against potential occupational health and safety risks. The extent to which safety in this field is a shared interest depends not only on the ideal-typical characteristics of the producer–consumer and the employer–employee relationship but also on relevant laws and regulations. Relevant to the producer–consumer relationship is the fact that under current product liability regulation in the EU, there is a strict liability for risks [67]. It prescribes caution and requires rigorous testing for products introduced to the market and holds companies liable for any undesirable effects of the products (in the case of normal use). For the employer–employee relationship, it is relevant that most Western countries have established a legal duty for employees to guarantee the safety of the work environment for employers by, for example, implementing risk management and preventative measures. This means that, as is the case with product liability, the health and safety of the risk-bearer (in this case, the employee) are a concern for both the risk-bearer and the party exposing that person to a risk (here, the employer). It is a shared interest according to the law.

However, the extent to which interests overlap is more encompassing in the case of the employer–employee relationship. Producers are primarily concerned with the safety of a specific product while employers are concerned with the health of personnel in their working environment. Thus, employers have a duty of care to their employees and, perhaps not surprisingly, the employer–employee relationship exhibits similarities to the more paternalistic doctor–patient relationship. The producer–consumer relationship, on the other hand, leaves much more room for personal consideration and is much more similar to an informative or consumerist relationship model (to use Emanuel and Emanuel’s typology). In general, we do not expect producers and commercial players to look beyond the safety of their products in meeting the needs those products are intended to meet. This would not be in the commercial interest of companies and would entail a level of engagement of producers with their clients that is rarely seen. Depending on the level of shared interest, it is to be expected that risk-bearer and risk-imposer are more engaged in supporting the risk-bearer’s decision-making strategy thereby enabling autonomous reflection.

### Room for Reflection

The relationship between employer and employee and that between producer and consumer vary in terms of dependency, strength of bonds, and level of shared interests. Since autonomy is shaped by the characteristics of relationships, the validity of informed consent is contingent on these characteristics. The discussion in this section suggests that employer–employee relationships and producer–consumer relationships provide different amounts of room for reflection. In working relationships, strong bonds with and dependency on the employer may limit opportunities for critical reflection and autonomous risk decisions. Conversely, in the producer–consumer relationship, room for reflection is particularly threatened by too loose bonds and the absence of shared interests. Thus, the framework developed in the previous section helps us to see that it might be hard to achieve valid informed consent in producer–consumer relationships and in employer–employee relationships albeit it for quite different reasons. This also means that what we can, and maybe should, do to improve the obtainment of valid informed consent is quite different for these different types of relationships.

## Discussion

In an ideal world, informed consent is given under optimal conditions of voluntariness, complete disclosure, and understanding of information. However, informed consent functions within the dynamics of a particular relationship between a risk-imposer and a risk-bearer. This relationship should preferably be symmetrical in order to decrease the dependency of the risk-bearer on the risk-imposer. A certain level of personal contact is needed to ensure proper information disclosure and to create a space for genuine discussion about the risks and benefits of a particular product in order to increase understanding. At the same time, the personal ties should not be so strong that the risk-bearer feels forced to act in accordance with the risk–imposer’s proposal without proper reflection—making it effectively a power relation. A strong overlap of the interests of the risk-imposer and the risk-bearer prevents conflicts of interests and deception or exploitation but some conflict of interest, or of values, between risk-imposer and risk-bearer may be instrumental in prompting reflection.

The world, however, is not perfect. Relationships between doctor and patient and between researcher and research participant, as well as those between producer and consumer and between employer and employee, differ in various ways from the ideal-typical relationship that is presupposed in informed consent. Dependency seems to be more asymmetrical in medical treatment and working relationships. The extent to which shared interests exist varies: doctor–patient relationships are typically characterized by strong overlapping interests, whereas this is less obviously the case for the other relationships. Furthermore, the proximity in these relationships is very different: Market relationships and research relationships (in particular biomedical relationships) may be rather impersonal and distant, whereas employees and employers may sometimes build upon long-term and often personal ties that are similar to those in treatment relationships. Of course, this characterization is based on a rather generalized image of what these relationships look like, and there may be many variations. There is a certain range, however, within which these relationships operate (Table 4).

**Table 4:** Overview of differences between relationships in which types of informed consent decisions play a role

Factor	Doctor–patient	Researcher–research participant		Producer–consumer	Employer–employee
		Biomedical research	Social science research		
Dependency of risk-bearer	High: great informational asymmetry; doctor is gatekeeper	Medium: great informational asymmetry, but participation in research is voluntary	Low: researcher depends on research participant for cooperation	Low	High: although employee may have some agency
Personal proximity	Strong: long-term	Weak: but with personal contact	Medium-strong	Very weak: distant and incidental	Strong: long-term and intensive
Shared interests	Substantial: health is shared interest	Limited	Medium: success of research is in interests of both	Limited: product safety	Substantial: safety is shared interest
Challenge to room for reflection	Informational and understanding asymmetry Too close bonds	Limited shared interest	Too proximate personal relationships	Absent or very distant ties	Power asymmetry

Different relationships pose different challenges when it comes to room for reflection and achieving valid informed consent. Much of the literature on informed consent identifies information dependency in the doctor–patient relationship as one of the major challenges in achieving informed consent. Our paper also argues that the close bonds in these relationships may also pose a risk to the capacity for reflection and autonomous choices. Highly proximate relationships leave little room for patient independence and may invoke paternalism. The relationship between employer and employee appears challenging in a different way. The hierarchy between them impedes the making of a genuinely voluntary decision. While there may be a degree of equality in the working relationship, the employee always depends on the employer to ensure the safety of the work environment and the security of her job. It is therefore unlikely that an employee will be in a completely symmetrical relationship with her employer which may threaten the voluntariness of risk decisions. In research relationships, and primarily in biomedical research relationships, the limited overlap in interests also creates problems for informed consent. There is often no immediate need for research participants to be part of a research project although history shows that deception has led to participation in research. In social science, the personal proximity poses more of a challenge.

Finally, the sheer distance between consumer and producer leads to informed consent being obtained rather indirectly. It is unlikely that producers will develop very strong ties with their consumers, and as a result, the risk-imposing producer cannot monitor whether the consumer has received and genuinely understands the information provided. Marketing departments could play a role in this^3^ but still the consumer can only consent through a market transaction and this hardly offers an opportunity to ask for further clarification. Consequently, different relationships provide different amounts of room for reflection both on the information available for making decisions and on the way such relationships shape the autonomy of the risk-bearer.

Considering the variations in these relationships, we conclude that informed consent would not function in the same way in different types of relationships. Institutional changes that may be useful in the doctor–patient relationship may have a detrimental effect in the employer–employee relationship and vice versa. In other words, any implementation of informed consent must take into account the particularities of the relationship in which the consent functions. Our systematic analysis is a first step toward improving the conceptualization of informed consent in a way that does justice to these relational differences by providing a more fine-grained account of the conditions under which valid informed consent can be obtained. The following subsection discusses the implications of this for the feasibility of obtaining informed consent for exposure to nanomaterial risks.

### Implications for Informed Consent as a Way to Deal with Nanomaterial Risks

Informed consent has been proposed as a way to deal with the risks and benefits that products containing nanomaterials pose to consumers. These efforts have typically focused on providing better information to consumers and on addressing the information asymmetry between producers and consumers [cf. 9]. Such efforts include, for example, the labeling of nano-content in cosmetics [68], additional product information, and the provision of online databases with voluntary application and risk information from the manufacturers [8, 20]. Both citizen panels and participatory risk assessment bring citizens’ knowledge level closer to that of developers [69] and give citizens influence on future technological developments [70]. NGOs often play a role in facilitating knowledge transfer, for instance by increasing the visibility of nanomaterial use and by encouraging informed public debate.

Although we know from experience with informed consent in doctor–patient relationships that it is important to address asymmetries in information and understanding, our analysis suggests that the weak ties between producers and consumers also present a major barrier to acquiring valid informed consent in producer–consumer relationships. These weak ties are a threat to the correct interpretation and understanding of information and may also result in very limited shared interest. Our findings suggest that if we want to strengthen informed consent in producer–consumer relationships, we should also focus on these aspects, rather than just on overcoming information dependency. This is, to some extent, already happening. NGOs do not only provide risk information but can also help establish relationships, for instance by starting a joint inquiry into the acceptability of risks [71]. Current trends in responsible innovation and design studies aim to create even closer ties between consumers and producers through means of participatory design and user-oriented design [72]. However, most consumer–producer relationships are still very distant, especially in an international context. Some argue that current developments in the field of nanotechnology create even more dependency from the global south to the global north [73].

When it comes to employer–employee relationships, we are not aware of attempts to explicitly use informed consent as a way to deal with nanomaterial risks to employees. Nevertheless, there are several ways in which European Occupational Health and Safety regulation promotes informed consent-like decision-making. For instance, EU Directive 89/391/EEC on the introduction of measures to encourage improvements in the safety and health of workers requires the provision of information, training of workers, and shared forms of decision-making (e.g., through consultation and participation). Something similar is prescribed in Council Directive 98/24/EC on the Protection against Chemicals Risks at Work. Such measures empower individual workers and ensure that information sharing becomes a mutual interest for both employer and employee.

More informal guidelines that have been developed to deal with nanoparticles at the workplace emphasize the need for direct communication between employer and employee [2, 4, 74]. The rationale behind this is that the uncertainty concerning nanomaterials risks has made the application of existing risk management strategies in workplaces particularly tricky. Conventional workplace exposure scenarios do not apply to nano-sized materials, and there is an absence of occupational exposure limits for most nanomaterials [1]. If employers do not know whether they can take adequate protective measures against uncertain risks, it seems reasonable to at least ask employees whether they mind being exposed to them. Yet, it must be noted that an obstacle is still the asymmetrical power relation between employers and employees which is also maintained in most Western labor laws. Here, labor unions play and have played a role in decreasing dependency of the employee on the employer [75, 76]. All in all, it seems that while the information component is increasingly addressed in employer–employee relationships, employees are often not in a formal position to make a decision regarding their own use of nanomaterials.

Much more research should be done on how the characteristics of relationships interact and could compensate for each other and facilitate room for reflection. A potentially more constructive, but speculative, approach to informed consent would be to see the three relational aspects we identified as communicating vessels. Mechanisms that point in this direction can already be observed. For instance, a high degree of dependency on employer’s decisions concerning the use of nanomaterials can to a certain extent be compensated for by a higher level of shared interests as is the case in labor law. More distant consumer relationships can also be partially compensated for by providing consumers with information about nano-content in their products as this decreases informational dependency. More research is needed to establish how these different relational factors could and should compensate for and interact with each other to achieve genuine informed consent for using, working with, and handling products containing nanomaterials. We hope that our discussion of informed consent provides a fruitful starting point for such an analysis.

## Footnotes

1 This paper focuses on nanomaterials that are intentionally produced as active nano-sized materials. We acknowledge that there is strong overlap in the toxic properties such materials may have with naturally occurring or process generated nanomaterials; therefore, much of the discussion that follows will be applicable to these materials as well.

2 In social scientific literature, the term social relations is often used to refer to more abstract notions of relationships, e.g., relations between classes and social groups. Personal relationships are more direct and often imply informal connections. This paper uses “relationship” to refer to relationships broadly construed, including both formal and information and distant and proximate relationships.

3 Developments such as the conscious consumerism movement show that consumers feel increasingly connected to certain brands on ethical grounds. This allows brands to build relationships with their costumers and establish a stable clientele.
